# ﻿Resurrection of *Euryadegeneri* (Pentaphylacaceae), endemic to Kaua‘i, Hawaiian Islands

**DOI:** 10.3897/phytokeys.249.131778

**Published:** 2024-11-15

**Authors:** J. Christopher Havran, Kenneth R. Wood, David H. Lorence

**Affiliations:** 1 Department of Biological Sciences, 205 Day Dorm Road, Campbell University, Buies Creek, North Carolina, USA Campbell University Buies Creek United States of America; 2 National Tropical Botanical Garden, 3530 Papalina Road, Kalāheo, Hawai‘i, USA National Tropical Botanical Garden Kalāheo United States of America

**Keywords:** Critically endangered, dioecious, *Eurya*, Hawaiian Islands, Kaua‘i, Pentaphylacaceae, resurrection

## Abstract

*Euryadegeneri* Kobuski (Pentaphylacaceae), a dioecious shrub endemic to Kaua‘i, is resurrected from synonymy with *E.sandwicensis* A. Gray. *Euryadegeneri* is distributed in montane mesic to wet forests in central and northwestern Kaua‘i. It can be distinguished from *E.sandwicensis* by its glabrous young stems, leaves and pedicels, linear-elliptic to ovate-elliptic leaves, longer pedicels (5–11 mm long), and shorter petals (4.5–7 mm long). A key to Hawaiian *Eurya* is provided. Two lectotypes are here assigned. *Euryadegeneri* is preliminarily designated as Critically Endangered according to the IUCN Red List guidelines.

## ﻿Introduction

*Eurya* Thunb. (Pentaphylacaceae) is a genus of approximately 130 species of dioecious shrubs and trees distributed across Asia and the Pacific (Wang et al. 2013). In the Hawaiian Islands *Eurya* has been documented from mesic to wet forests on the islands of Kaua‘i, O‘ahu, Moloka‘i, Maui, and Hawai‘i (Wagner et al. 1999). Kobuski (1935) recognized two species and three infraspecific taxa of Hawaiian *Eurya*. The two species were differentiated by leaf shape: *E.degeneri* with cuneate leaf bases and acute apices and *E.sandwicensis* with subcordate to truncate leaf bases and obtuse or rounded apices. Wagner et al. (1999) placed all taxa described by Kobuski into synonymy with *E.sandwicensis*. Over the past several decades the National Tropical Botanical Garden (NTBG) Science staff have rigorously studied and documented the distribution and abundance of Hawaiian *Eurya*. Careful examination of stems, leaves, and floral structures from these more recent collections reaffirm Kobuski’s (1935) designation of *E.degeneri* as a species and have revealed additional characters that further distinguish species of Hawaiian *Eurya*. In addition to the leaf shape characteristics described by Kobuski (1935), *E.degeneri* differs from *E.sandwicensis* in possessing glabrous young stems, leaves, and pedicels. Flowers in *E.degeneri* also have longer pedicels and shorter petals than *E.sandwicensis*. To date, no plants fitting the description of *E.degeneri* have been documented outside of Kaua‘i.

## ﻿Methods

Herbarium specimens from PTBG were examined for the description of species. Specimens were selected to demonstrate the range of observed morphological variation and geographic distribution of *Eurya* across Kaua‘i. Measurements for vegetative traits were made to the nearest mm. Both male and female flowers were selected for examination across both species. Flowers were rehydrated by boiling and measurements for reproductive traits were made to the nearest 0.5 mm. Herbarium specimens and label data were referenced for estimation of flowering phenology of *E.degeneri* and *E.sandwicensis* on Kaua‘i. Diacritical marks following Pukui et al. (1974) were added to place names in the Specimens Examined sections. The proposed conservation status articulated here for *E.degeneri* follows the IUCN Categories and Criteria (IUCN 2012) and guidelines of the IUCN (IUCN 2024).

## ﻿Taxonomic treatment

### ﻿1. Eurya degeneri

Taxon classificationPlantaeEricalesPentaphylacaceae

Kobuski, J. Arnold Arbor. 16: 351. 1935.

5880532F-FE30-5B50-ABF7-BC4FC804A5FA

Figs 1 , 3A

Eurya sandwicensis A. Gray forma grandifolia Wawra, Flora. 56: 168. 1873. Type: Hawaiian Islands. Kaua‘i: *Wawra 2025* (lectotype, designated here: W [W0221862!]; isolectotype: W [W0221863!]).
Eurya degeneri Kobuski forma grandifolia (Wawra) Kobuski, J. Arnold Arbor. 16: 352. 1935. Type: Based on E.sandwicensisA. Grayvar.grandifolia Wawra.
Eurya degeneri Kobuski forma stenophylla Kobuski, J. Arnold Arbor. 16: 352. 1935. Type: Kaua‘i, *Rock 17274* (holotype: P [P00641578!]).

#### Type material.

**USA** • **Hawaiian Islands.** Kaua‘i: Open forest, Waineke swamp Koke‘e, 1 Jul, 1926, *O. Degener 8675* (holotype: A [00024935!]; isotypes: BISH [BISH1014632!, BISH1014633!], NY [NY0035824!], WIS [0255833!]).

**Figure 1.**
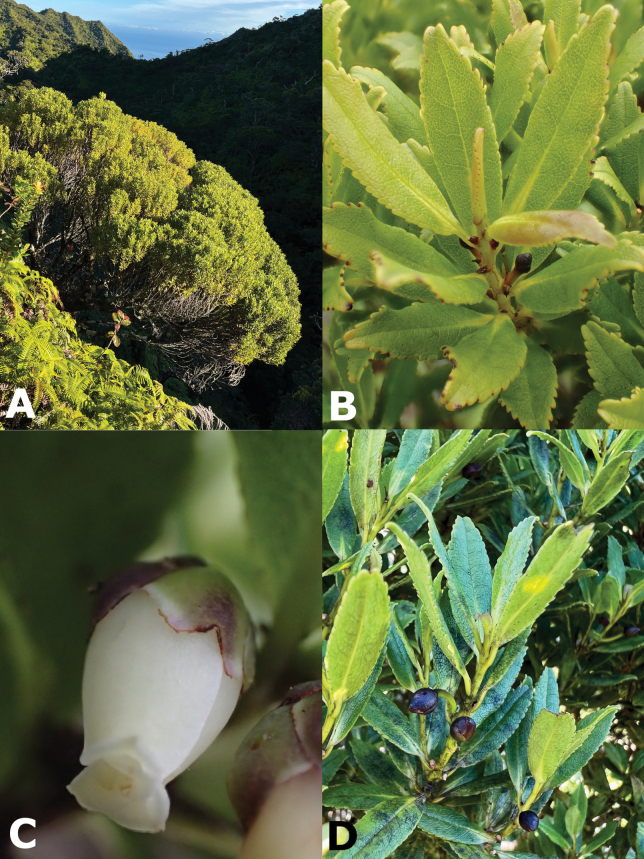
*Euryadegeneri* Kobuski **A** habit **B** leafy twig **C** flower **D** fruits. Photos by K.R. Wood. **A, B, D***Wood, Heintzman & Deans 18866* (PTBG) **C***Wood & Perlman 17935* (PTBG).

#### Description.

Densely branched shrubs or trees 1.5–5(–7) m tall, young stems glabrous, rarely glabrate. ***Leaves*** closely spaced; blades coriaceous, linear-elliptic, rarely oblong-elliptic, (1.5–) 2.2–5.0(–9.0) cm long, 0.7–2.4(–3.0) cm wide, midrib tinged reddish, secondary veins yellow-green, lower surface conspicuously reticulate, glabrous, upper surface glabrous, margins weakly revolute, crenulate with black inflexed mucronate teeth, apex acute to narrowly acute, occasionally acuminate, base acute to cuneate, rarely rounded, petioles 2–4 mm long. ***Flowers*** 1, rarely 2 in the leaf axils, erect to occasionally nodding; pedicels 5–11 mm long, glabrous, rarely glabrate; bracteoles 2, minute; sepals 5, purplish brown, thick-coriaceous, suborbicular, unequal, 3–6 mm long, glabrous, rarely glabrate, margins scarious, persistent, enlarging up to 6 mm in fruit; petals 5, pale yellow to cream, somewhat fleshy, obovate, 4.5–7 mm long, connate at base; staminate flowers with 10 stamens, filaments distinct, 1.5 mm long, adnate to base of petals, ca. 1/2 as long as anthers, anthers opening by longitudinal slits; pistillate flowers with 8–10 staminodes, staminodes 2–3.5 mm long, ovary 3-celled, styles 3, 1.5–2 mm long. ***Fruit*** a globose berry, dark bluish black, 7 mm in diameter. Seeds numerous.

#### Etymology.

The specific epithet was selected by Kobuski (1935) to honor Otto Degener, whose collections in *Eurya* informed taxonomic work in the genus.

#### Phenology.

*Euryadegeneri* has been collected with buds, flowers, and fruit from February through October.

#### Conservation status.

IUCN Red List Category. *Euryadegeneri* demonstrates an Extent of Occurrence (EOO) of ca. 80 km^2^, an Area of Occupancy (AOO) of 34 km^2^, and a population of ca. 217 mature individuals fragmented across 10 subpopulations. National Tropical Botanical Garden researchers have observed declines in EOO, AOO, habitat quality, and number of individuals in subpopulations of *E.degeneri*. When these population characteristics are evaluated using the World Conservation Union’s IUCN (2012) Red List Categories and Criteria *Euryadegeneri* is preliminarily designated as: CR B1ab(i,ii,iii,iv,v)+ C2a(i). This Critically Endangered (CR) category designates the species as facing a very high risk of extinction in the wild. Relevant threats to both species of Hawaiian *Eurya* are elaborated upon in the “Conservation” section below.

#### Specimens examined.

**USA** • **Hawaiian Islands.** Kaua‘i: Alaka‘i bog near Kilohana lookout, 1210 m, 13 Feb 2004, *K.R. Wood & T. Menard 10574* (PTBG, BISH, US) • Hanākapī‘ai headwaters, along crown ridge between Pihea and Wainiha, 1250 m, 22 Jul 2008, *K.R. Wood 13187* (PTBG, BISH, US) • Awa‘awapuhi, above stream and south of Kainamanu, 1088 m, 7 Feb 1995, *K.R. Wood & D. Boynton 4022* (PTBG) • Kalalau Rim, North, below Puu o Kila, 950–1150 m, 6 Jul 1991, *K.R. Wood 1022* (PTBG) • Limahuli Preserve, rim of Wainiha between Hono o Nā Pali and Pali ‘Ele‘ele, 975 m, 14 Oct 2007, *K.R. Wood & N. Tangalin 12585* (PTBG, BISH) • Limahuli Valley, east side of ridge separating Limahuli and Hanākapī‘ai Valleys, above major waterfall, 770 m, 6 Aug 1991, *K.R. Wood et al. 1109* (PTBG, MO, F) • Ridge below upper weather port, Hanākapī‘ai side, 888 m, 30 Jun 2015, *S. Walsh & M. Edmonds SKW78* (PTBG) • Upper Limahuli Preserve, Hanākapī‘ai side in upper back bowl, 951 m, 19 Nov 2011, *N. Tangalin 2898* (PTBG, NY, UC) • Hono O Nā Pali NAR, Pōhākea region, steep ridge above Hanākapī‘ai, 914 m, 28 Jul 2011, *K.R. Wood & M. Query 14733* (PTBG, BISH, MO, NY, US) • Hanākapī‘ai, 1100 m, 14 Jan 2011, *K.R. Wood et al. 18866* (PTBG) • ‘Ohi‘a/‘Ōlapa Forest, 968 m, 5 Mar 2019, *A.M Williams AMW559* (PTBG) • Kalalau valley, back of valley below Pihea area, 762 m, 15 Jun 2000, *K.R. Wood & D. Boynton 8541* (PTBG) • Ridge down valley from near Pihea, 1054 m, 23 Jul 1992, *S. Perlman and K.R. Wood 12905* (PTBG) • Kokee, Hono O Nā Pali NAR, Pihea Trail to Pihea Vista and out east along ridge, 1321 m, 27 Sep 2010, *N. Tangalin & K.R. Wood 2403* (PTBG, BISH, NY) • Lā‘au Ridge, plateau region north of Pu‘u Kamana and south of Kamakeanu, 1250 m, 17 Feb 2000, *K.R. Wood et al. 8242* (PTBG) • Limahuli, upper east drainage between Pali ‘Ele‘ele and Hono O Nā Pali, 914 m, 9 Oct 1996, *K.R. Wood & S. Perlman 5696* (PTBG, BISH, US) • North Bog, Rim of Wainiha, 1189 m, 29 Feb 2000, *K.R. Wood et al. 8285* (PTBG) • Waikoali stream, Alaka‘i Swamp, stream banks, 1127 m, 12 Jun 1996, *S. Perlman & K.R. Wood 15400* (PTBG) • Waimea District, Koke‘e State Park, along Kawaikōī Stream Trail, about 0.5 km northeast of intersection with Pihea Trail, 1010 m, 8 Sep 1988, *D.H. Lorence et al. 6273* (PTBG) • Waikoali Bogs, Circle Bog, and scattered bogs to NE, 1219–1280 m, 21 Nov 1994, *K.R. Wood et. al 3777* (PTBG) • Mohihi, 1183 m, 20 Sep 2018, *K.R. Wood & S. Perlman 17935* (PTBG).

### ﻿2. Eurya sandwicensis

Taxon classificationPlantaeEricalesPentaphylacaceae

A. Gray, Bot. U.S. Expl. Exped. 1839–1842. 1: 209. 1854.

A9190030-A5CD-57FC-8E1A-35065F5C517A

Figs 2 , 3B

Ternstroemiopsis sandwicensis (A. Gray) Urb., Ber. Deutsch. Bot. Ges. 14: 49. 1896. Based on Euryasandwicensis A. Gray.
Eurya sandwicensis A. Gray var. prostrata Kobuski, J. Arnold Arbor. 16: 350. 1935. Type: Hawaiian Islands. Moloka‘i, *Degener 8676* (lectotype (designated here): A [00024940!]; isolectotype: A [00024939!]; isotypes: BISH [BISH1014635, BISH1014636!], G [G00354796!], MASS [00320484!], NY [00353842!], US [00435645!], WIS [0255835!]).

#### Type.

**USA** • **Hawaiian Islands.** O‘ahu: *W. Rich s.n.* (lectotype GH [00024938!], designated by St. John (1985: 569); isolectotypes: NY [00353841!], P [P00641573!], US [00113988!].

**Figure 2.**
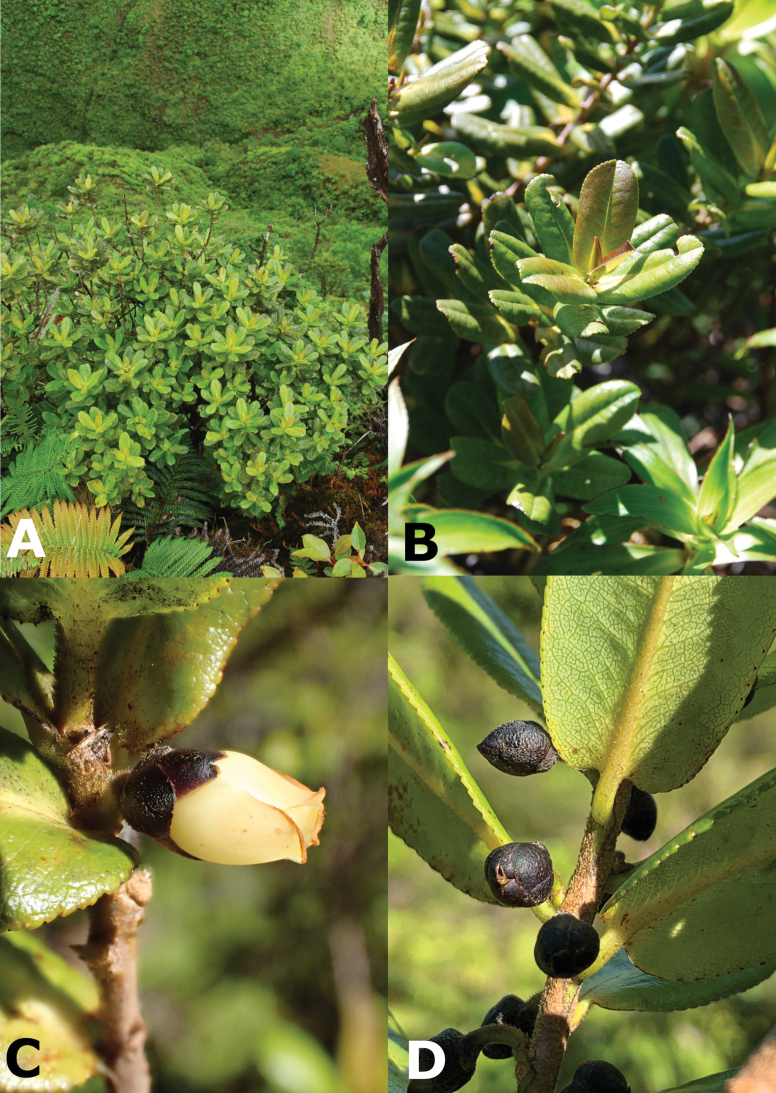
*Euryasandwicensis* A. Gray **A** habit **B** leafy twig **C** flower **D** fruits. Photos by K.R. Wood **A***Wood, Query & Perlman 16077* (PTBG) **B***Wood, Query & Perlman 16187* (PTBG) **C, D***Wood, Heintzman & Deans 18839* (PTBG).

#### Description.

Moderately branched shrubs or trees 1.5–4(–6) m tall, young stems sericeous to strigose, rarely glabrate, hairs golden yellow. ***Leaves*** closely spaced; blades coriaceous, ovate to oblong, rarely obovate, (2.5–)3.0–6.5(–9) cm long, (1.2–)1.5–3.0(–4) cm wide, midrib tinged reddish, secondary veins yellow-green, lower surface conspicuously reticulate, strigose along midrib, sparsely so on secondary veins, upper surface glabrous to glabrate, margins weakly revolute, crenulate with black inflexed mucronate teeth, apex obtuse to rounded, rarely acute or emarginate, base subcordate to truncate, rarely cuneate; petioles 1–3(–4) mm long. ***Flowers*** 1, rarely 2 in the leaf axils, nodding to suberect; pedicels 3–5(–7) mm long, ± strigose with golden hairs; bracteoles 2, minute; sepals 5, purplish brown, thick-coriaceous, suborbicular, unequal, 3–7 mm long, ± strigose, margins scarious, persistent, enlarging up to ca. 8 mm long in fruit; petals 5, pale yellow to cream, somewhat fleshy, obovate, (5–)8.0–10.5 mm long, connate at base; staminate flowers with (10–)15–16 stamens, filaments distinct, 1–2 mm long, adnate to base of petals, ca. 1/2 as long as anthers, anthers opening by longitudinal slits; pistillate flowers with (5–)8–10 staminodes, staminodes 2–3.5 mm long, ovary 3- or rarely 4-celled, styles 3 or rarely 4, 1.5–2 mm long. ***Fruit*** a globose berry, dark bluish black, 7–10 mm in diameter. Seeds numerous.

**Figure 3.**
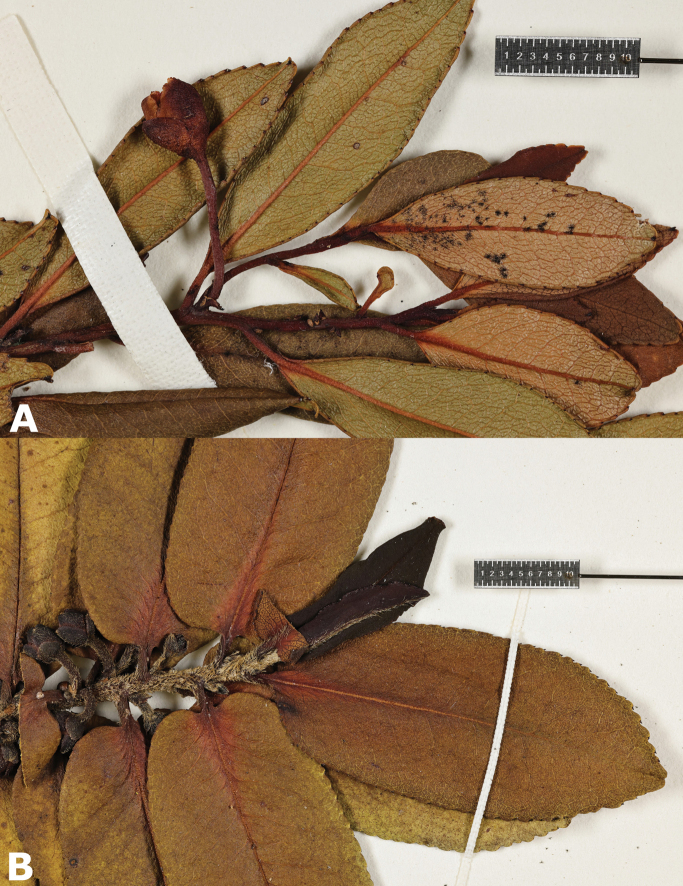
Leaf and stem comparison of Kaua‘i species of *Eurya***A***Euryadegeneri***B***Euryasandwicensis*. Photos by Neil Brosnahan **A***Wood, Perlman & Mehrhoff 3777* (PTBG) **B***Wood, Kirkpatrick & Perlman 15599* (PTBG).

#### Etymology.

The specific epithet was selected by Asa Gray (1854) to acknowledge the former European name of the Hawaiian Islands.

#### Phenology.

On Kaua‘i, *Euryasandwicensis* has been collected with flowers from January through September and with fruit from April through December.

#### Conservation status.

The conservation status of *Euryasandwicensis* was last assessed in 1998. At that time, it was listed as Vulnerable (World Conservation Monitoring Centre 1998).

#### Specimens examined.

**USA** • **Hawaiian Islands.** Kaua‘i: Alaka‘i, east of the Sincock bog system, wet forest near Kapoki, 1426 m, 8 May 2007, *K.R. Wood & J. Fafson 12371* (PTBG) • Alaka‘i Swamp, Kaua‘i Forest Bird Survey, transect 3 between Halepaakai and Halehaha streams, 14 Feb 1989, *S. Perlman et al. 10630* (PTBG, US, BISH, MO) • Along ridge between Kāhili and Kawaikini, back of Hanapēpē valley on one side and ‘Iole on the other, 1116–1189 m, 19 Sept 1994, *S. Perlman & K.R. Wood 14389* (PTBG) • Hanalei District, Alakai, 1459 m, 8 Feb 2012, *K.R. Wood 14878* (PTBG, UC) • Summit of Nāmolokama, 1265 m, 1 Feb 2000, *K.R. Wood 8171* (PTBG) • ‘Iole headwaters, 991 m, 10 Jan 2012, *K.R. Wood 14824* (PTBG) • *loc. cit.*, 853 m, 28 Aug 2013, *K.R. Wood et al. 15653* (PTBG, BISH, CAS) • *loc.cit*., 870 m, 8 Aug 2013, *K.R. Wood et al. 15599* (PTBG, BISH) • *loc.cit*., 890 m, 29 Jul 2021, *K.R. Wood et al. 18789* (PTBG) • *loc.cit*., 890 m, 20 Oct 2021, *K.R. Wood et al. 18839* (PTBG) • Kamo‘oloa headwater drainage below Kapalaoa, [853–975 m], 4 Oct 1996, *K.R. Wood 5669* (AD, PTBG) • Wahiawa headwaters, SE below Kapalaoa, 863 m, 22 Dec 2010, *K.R. Wood & N. Tangalin 14442* (PTBG) • NE Alaka‘i, 1400 m, 13 Aug 2018, *K.R. Wood 17905* (PTBG, BISH, CAS) • Ridge north of Kāhili, between Kapalaoa and Kawaikini, 963 m, 20 Sept 1994, *S. Perlman & K.R. Wood 14395* (PTBG, HAST) • Ridge south of Kawaikini, 1400 m, 18 Sep 2014, *K.R. Wood, M. Query, & S. Perlman 16077* (PTBG) • Ridge south of Kawaikini, 1341 m, 7 Jan 2015, *K.R. Wood & M. Query 16187* (PTBG) • Wahiawa Mtns, on the ridge between Mt. Kāhili and the relay towers, 30 Aug 1984, *T. Flynn 937* (PTBG) • Waimea Canyon, 792 m, 11 Apr 2010, *K.R. Wood 14182* (PTBG, BISH, US).

##### ﻿Key to the Hawaiian species of *Eurya*

Distinguishing features of the two species of Hawaiian *Eurya* are summarized in Table 1. The species can be identified with the following key.

**Table:** 

1	Young stems glabrous; leaf blades linear-elliptic to oblong-elliptic, lower leaf surface glabrous, leaf apex acute to narrowly acute, leaf base acute to cuneate; pedicels 5–11 mm long, glabrous; petals 4.5–7.0 mm long; sepals glabrous, rarely glabrate	**1. *Euryadegeneri***
–	Young stems sericeous to strigose; leaf blades ovate to oblong, lower leaf surface strigose along midrib, sparsely so on secondary veins, leaf apex obtuse to rounded, leaf base subcordate to truncate; pedicels 3–5 mm long, strigose; petals (5–)8.0–10.5 mm long; sepals ± strigose	**2. *Euryasandwicensis***

##### ﻿Relationships within Hawaiian *Eurya*

Although morphologically distinct and with different distributions, *E.degeneri* and *E.sandwicensis* share similar habitats, conservation threats, and reproductive traits. We describe and compare morphology, distribution, ecology, conservation, and reproductive attributes for the two Hawaiian *Eurya* species in the subsequent passages.

###### ﻿Morphology

*Euryadegeneri* can be distinguished from *E.sandwicensis* by its densely branched habit (*vs* moderately branched); having young stems glabrous (*vs* sericeous to strigose); leaf blades linear-elliptic to ovate-elliptic (*vs* ovate to oblong); lower leaf surface glabrous (*vs* strigose along midrib, sparsely so on secondary veins); leaf apex acute to narrowly acute (*vs* obtuse to rounded); leaf base acute to cuneate (*vs* subcordate to truncate); pedicels 5–11 mm long, glabrous (*vs* 3–5 mm long, strigose); and petals 4.5–7.0 mm long (*vs* (5–)8.0–10.5 mm long in *E.sandwicensis*) (Table 1).

**Table 1.** Comparison of morphological characters for Hawaiian species of *Eurya*.

Character	*E.degeneri*	*E.sandwicensis*
Habit	densely branched shrub or tree	moderately branched shrub or tree
Young stems	glabrous	sericeous to strigose
Leaf blade	linear-elliptic to ovate-elliptic	ovate to oblong
Lower leaf surface	glabrous	strigose along midrib, sparsely so on secondary veins
Leaf apex	acute to narrowly acute	obtuse to rounded
Leaf base	acute to cuneate	subcordate to truncate
Pedicels	5–11 mm long, glabrous	3–5 mm long, strigose
Petals	4.5–7.0 mm long	(5–)8.0–10.5 mm long
Sepals	glabrous, rarely glabrate	strigose

###### ﻿Distribution

**Figure 4.**
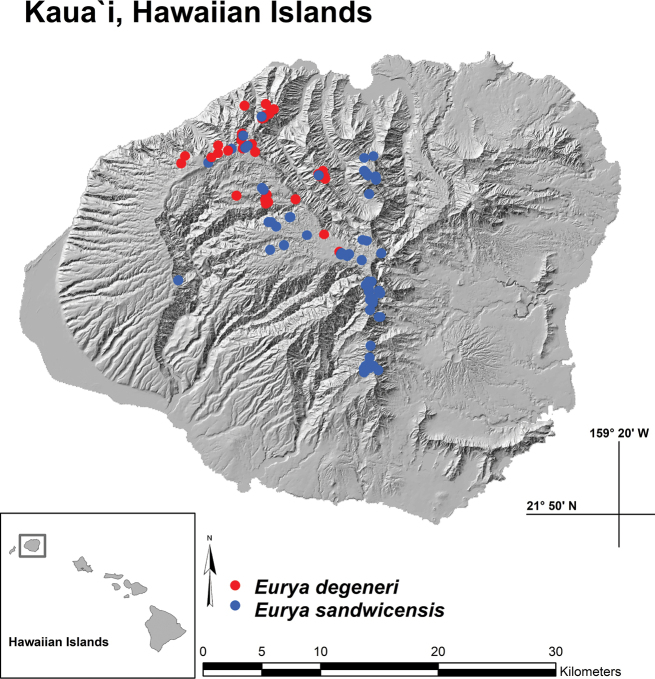
Distribution map of known locations of *Euryadegeneri* and *E.sandwicensis* on Kaua‘i.

*Euryadegeneri* is known from ca. 217 individuals distributed around the central to northwestern regions of Kaua‘i. They occur in ten fragmented sub-populations, having an extent of occurrence (EOO) of ca. 80 km^2^, an area of occupancy (AOO) of ca. 34 km^2^, with an elevational range of 762–1585 m (Fig. 4). *Euryasandwicensis* has a multi-island distribution, including the islands of Kaua‘i, O‘ahu, Moloka‘i, Maui, and Hawai‘i. *Euryasandwicensis* is only known from 600–700 individuals on Kaua‘i and is even rarer on the other islands. Although *E.sandwicensis* and *E.degeneri* overlap in their distribution in the central and northwestern regions of Kaua‘i, the two taxa have never been observed growing together. Colonies of *E.sandwicensis* also extend down along the northern and southern windward ridges of Kaua‘i where *E.degeneri* is not found. The constrained range of *E.degeneri* suggests that this taxon has a more restricted range of ecological parameters to which it is suited.

###### ﻿Ecology

Both *Euryadegeneri* and *E.sandwicensis* predominantly occur in *Metrosideros* Banks ex Gaertn. (Myrtaceae) / *Cheirodendron* Nutt. ex Seem. (Araliaceae) montane wet forests and are often associated with a mix of other tree genera such as *Polyscias* J.R. Forst. & G. Forst. (Araliaceae), *Dubautia* Gaudich. (Asteraceae), *Perrottetia* Kunth (Dipentodontaceae), *Cyrtandra* J.R. Forst. & G. Forst. (Gesneriaceae), *Antidesma* L. (Phyllanthaceae), *Hydrangea* Gronov. (Hydrangeaceae), *Syzygium* Gaertn. (Myrtaceae), *Bobea* Gaudich., *Coprosma* J.R. Forst. & G. Forst., *Kadua* Cham. & Schltdl., *Psychotria* L. (all Rubiaceae), and *Melicope* J.R. Forst. & G. Forst. (Rutaceae). Terrestrial matting ferns such as *Dicranopteris* Bernh., *Diplopterygium* (Diels) Nakai, and *Sticherus* C. Presl (all Gleicheniaceae) are also common components along with sedges such as *Gahnia* J.R. Forst. & G. Forst., *Carex* L., and *Machaerina* Nees (all Cyperaceae). Both species of *Eurya* on Kaua‘i are found growing in highly weathered soils having low fertility (Deenik and McCellan 2007).

###### ﻿Conservation

Relevant threats to the habitat of both species of *Eurya* on Kaua‘i include feral pigs (*Susscrofa*), rats (*Rattus* spp.), and occasional feral goats (*Caprahircus*) and black-tailed deer (*Odocoileushemionus*). Landslides after heavy rains can also be a very serious threat, especially the steeper habitats along the windswept ridges and bases of cliffs. The most serious invasive non-native plant species include *Miconiacrenata* (Vahl.) Michelang. (Melastomataceae), *Sphaeropteriscooperi* (Hook. ex F. Muell.) R.M. Tryon (Cyatheaceae), *Buddlejaasiatica* Lour. (Scrophulariaceae), *Psidiumcattleyanum* Sabine (Myrtaceae), *Cyperusmeyenianus* Kunth (Cyperaceae), and *Hedychiumgardnerianum* Sphep. ex Ker Gawl. (Zingiberaceae).

###### ﻿Reproductive biology

Although the genus *Eurya* is described as dioecious or rarely monoecious (Wagner et al. 1999; Min and Bartholomew 2007), Wang et al. (2013) report that hermaphroditic flowers were observed on *E.obtusifolia* H.T. Chang, thereby expanding our understanding of the breeding systems of *Eurya*. For Hawaiian *Eurya*, most flowers we examined were unisexual with plants dioecious, but it should be noted that several flowering voucher specimens in this study appeared to have perfect flowers (i.e., *E.sandwicensis* (*Perlman et al. 10630* (PTBG, US), *Wood 14878* (PTBG, UC) and *E.degeneri* (i.e., *Wood & Boynton 4022* (PTBG)). In addition, some *E.degeneri* specimens in northwestern Kaua‘i demonstrate leaf characteristics that are large for the species (i.e. *Perlman & Wood 12905* (PTBG), *Walsh & Edmonds SKW78* (PTBG, MBK), and *Wood et al. 1109* (PTBG, MO, F)). This large-leaved morphotype was the basis for an infraspecific taxon (E.degenerif.grandifolia (Wawra) Kobuski), but as these individuals are distributed in the same geographic region as *E.sandwicensis*, they may represent hybrids between the two species. Further research is needed to determine if some flowers on Hawaiian *Eurya* can be truly perfect with functional stamens and pistils and if hybridization can occur between the two taxa on Kaua‘i.

## Supplementary Material

XML Treatment for Eurya degeneri

XML Treatment for Eurya sandwicensis

## ﻿Additional information

### Author ORCIDs

J. Christopher Havran https://orcid.org/0000-0003-3475-679X

Kenneth R. Wood https://orcid.org/0000-0001-6446-1154

David H. Lorence https://orcid.org/0000-0002-6735-9531

### Data availability

All of the data that support the findings of this study are available in the main text.
